# The mouse to man conundrum: Revisiting an old lung injury model to study repurposed drugs for COVID‐19 treatment

**DOI:** 10.1113/EP091889

**Published:** 2024-04-12

**Authors:** Andreas Ronit, Ronni R. Plovsing

**Affiliations:** ^1^ Department of Infectious Diseases Copenhagen University Hospital – Amager and Hvidovre Hospital Hvidovre Denmark; ^2^ Department of Anaesthesiology and Intensive Care Copenhagen University Hospital – Amager and Hvidovre Hospital Hvidovre Denmark; ^3^ Department of Clinical Medicine, Faculty of Health and Medical Sciences University of Copenhagen Copenhagen Denmark

**Keywords:** acute respiratory distress syndrome, COVID‐19, immunology, lung injury model

1

The pathophysiology of acute respiratory distress syndrome (ARDS) is complex and the mechanisms of injury are many, both in the lung and systemically, but nevertheless it leads to acute hypoxaemic respiratory failure with a high mortality rate owing to diffuse lung inflammation and alveolar influx of protein‐rich fluid. Various disorders, either direct or indirect, can lead to ARDS, with the most common being pneumonia and non‐pulmonary sepsis, whereas transfusion‐related acute lung injury and ischaemia–reperfusion injury after thoracic surgery are among the rarer causes of ARDS. On that note, early case reports suggested an association between development of ARDS in patients who underwent thoracic procedures within months after treatment with the anti‐neoplastic agent bleomycin (Goldiner, 1978). This was attributed, in part, to free radical‐promoting ability of bleomycin and exposure to high oxygen concentrations during anaesthesia (Berend, 1984), but larger studies have failed to demonstrate an association between perioperative oxygen supply and ARDS in bleomycin‐treated patients (Aakre et al., 2014). Although the pathophysiological relevance of bleomycin in clinical ARDS can be questioned, the bleomycin model remains a commonly used model of acute lung injury in mice. Developed in the 1970s as an animal model for experimental lung fibrosis, it uses a single dose of bleomycin, often delivered intratracheally, and reproduces the overall inflammatory pattern of ARDS, with early exudation and influx of inflammatory cells, including neutrophils, and subsequent development of fibrosis (Matute‐Bello et al., 2008).

In this issue of *Experimental Physiology*, Meunier et al. (2024) studied the effects of antibodies antagonizing interleukin (IL)‐1 and IL‐6 receptors (including tocilizumab) in mice treated with intranasal bleomycin. The authors were not able to identify beneficial effects on lung tissue, alveolocapillary barrier integrity or neutrophil influx, and no reduction in mRNA expression of inflammatory mediators (tumor necrosis factor‐α, IL‐6, IL‐8 and monocyte chemoattractant protein‐1) was observed. These outcomes were all assessed on day 7. Although bleomycin induced diffuse lung injury in the vast majority, <20% of total lung area was affected, and none of the animals experienced respiratory failure or death attributable to bleomycin, which does not seem to represent the clinical consequences of human ARDS.

Plasma and bronchoalveolar levels of several cytokines, including IL‐6, have been identified as one of many biomarkers of lung injury and are also associated with morbidity and mortality in coronavirus disease 2019 (COVID‐19)‐associated ARDS. However, whether the increased bronchoalveolar fluid levels of IL‐6 observed in ARDS are causally associated with lung injury or a byproduct or prevent lung injury remains controversial, and as an example of this complexity, IL‐6 seems to be crucial for both protection and lung repair in models of lung injury caused by influenza (Dienz et al., 2012). Thus, although the authors claimed that bleomycin failed to alleviate inflammation, blocking IL‐6 could, on the contrary, also have worsened lung injury and delayed subsequent repair. Given that inflammation is not a linear process, and with the uncertainty (i.e., timing) regarding when to block or not, sampling at earlier and later time points could have provided further insight.

The authors previously studied dexamethasone, a potent corticosteroid, in a similar model, which also failed to alleviate bleomycin‐induced lung injury (Vega et al., 2019). As with IL‐6, the pulmonary effects of dexamethasone in ARDS are complex and not completely understood. In a previous clinical study, we were not able to show differential gene expression of inflammatory mediators, including tumor necrosis factor‐α, IL‐6, IL‐8 and monocyte chemoattractant protein‐1, in bronchoalveolar fluid of patients with COVID‐19 ARDS treated with dexamethasone versus not receiving corticosteroids (Fahnøe et al., 2022). Both IL‐6 and dexamethasone have diverse functions in regulating the innate and adaptive immune system, and it could therefore be relevant to include additional outcome measures in future studies, such as a more complete immune transcriptomic profile and functional studies of the development of fibrosis. Another relevant intervention to study in these models could be CXCL1‐receptor antagonists, with CXCL1 being the functional homologue of human IL‐8 (a potent neutrophil attractant and activator), which might precede development of ARDS and seems to be of particular importance in COVID‐19 ARDS. This could be coupled to more detailed analyses of neutrophil recruitment and activation, in addition to transcompartmental flux of immune cells and cytokines.

Recent clinical trials on the early use of dexamethasone in both classical and COVID‐19 ARDS and IL‐6 receptor blocking (although with variable efficacy of tocilizumab between trials) have showed improved survival in COVID‐19 ARDS (Recovery Collaborative Group, 2021; REMAP‐CAP Investigators, 2021). A key remaining question nonetheless seems to be ‘how’ these drugs exert their beneficial effect rather than ‘if’ in a heterogeneous clinical setting, because their effect on lung pathophysiology and immunity is not understood. Mouse is not man, but man should still learn from experimental models of ARDS that allow for a much more invasive approach and testing of hypotheses and combinations of treatment or new drugs or, as in this case, reveal information about how repurposed drugs, such as dexamethasone and tocilizumab, might exert their effect in the lungs and thus serve to bridge the gap owing to the inherent challenges of mechanistic studies in humans.

## AUTHOR CONTRIBUTIONS

Both authors contributed equally to the writing of the manuscript.

## CONFLICT OF INTEREST

None declared.

## FUNDING INFORMATION

None.
